# Strategies for Improvement Quality of Life in Menopause

**DOI:** 10.5812/nms.10819

**Published:** 2013-06-27

**Authors:** Masoumeh Abedzadeh Kalahroudi

**Affiliations:** 1 Trauma Nursing Research Center, Kashan University of Medical Sciences, Kashan, IR Iran

**Keywords:** Menopause, Quality of Life, Improvement

## 1. Introduction

Menopause is a universal experience for women that occur, in around 50 years old. In this periods women encounter with many changes in their life. Retirement and children leaving home can cause the loneliness and depression, however this time is an opportunity for women to changing their life. The women perception of menopause depends on their social, cultural, economical status and life style factors. Many sign and symptoms such as hot flash, night sweats, lack of energy, anxiety , nervousness ,sleep disturbance, change in sexual desire and avoiding intimacy is experienced by women in this periods and affects their health status (1).

## 2. Arguments

In the 21st century, promotion of the quality of life is considered as one of the goals of health care services for all the people. Based on WHO definition quality-of-life is "individual's perception of their position in life in the context of the culture and value systems in which they live and in relation to their goals, expectations, standards and concerns" (2).

In menopausal period, quality-of-life usually refers to the vasomotor and sexual symptoms. These signs affect the physical and mental health, life satisfaction, and finally quality of life.

With increasing the proportion of elderly population in worldwide, the number of women who live one–third of their life in a hypo estrogenic status will be increased. Therefore improvement of quality of life in menopause is an important issue. Evidence shows that the quality of life decreases during menopause (3, 4). Some of the demographical and life style factors such as educational level, employment status, physical activities ,income satisfaction, duration of menopause , marriage satisfaction and the number of children living with family are related with quality of life in menopausal women (5, 6).

Influencing on some of these factors such as educational level, employment status, financial situation and other individual factors affecting the quality of life is very difficult. Therefore should be emphasized on education, life style modification, social and family supports. 

Planning the educational programs and increasing awareness of women regarding menopause will improve the women's attitude towards this phenomenon and increases their health behavior and ultimately lead to improve their quality of life. 

Since most women in Iran have access to health care centers, it seems most appropriate and accessible strategies for improving women's quality of life is training classes and consulting about menopause problems with a health promotion approach (7).

Menopause is an opportunity for women to modify their lifestyle with regular physical activity. Evidence shows that physical activity has a positive effect on symptoms of menopause especially hot flash and subsequently on quality of life (6, 8). Dietary habits are another component of life style and its modification is very important. Evidence shows that intake of soy products (Isoflavones) have an influence on the incidence and severity of menopausal symptoms and therefore affect the quality of life (9).

## 3. Conclusion

Perception of symptoms and problems of menopause and support by family, especially by the spouse can have a positive role in improving women's mental condition. Establishment of social support networks and programs to promote physical and mental health helps to improve the quality of life in menopausal women (7).

Finally since the menopause impacts social, emotional, physical and sexual aspects of quality of life, therefore a holistic management with special attention to the non-pharmacological methods is required. Now I invite all specialists to a discussion on how a holistic approach including using complementary and alternative medicine can affect the quality of life after menopause.
